# Microleakage of zinc oxide eugenol, resin, calcium hydroxide, bioceramic and bioactive glass sealers using stereomicroscope: An *in vitro* study

**DOI:** 10.6026/973206300223723

**Published:** 2026-06-30

**Authors:** Shivani Rawat, Anu Narang, Santosh Kumar Singh, Gayatri H. Giramkar, Sadhvi Pateriya, Naman Keshwani

**Affiliations:** 1 Department of Conservative Dentistry and Endodontics, People's College of Dental Sciences and Research Centre, People's University, Bhopal, Madhya Pradesh, India

**Keywords:** Microleakage, root canal sealers, bioactive glass, bioceramic sealer, dye penetration

## Abstract

Microleakage is a major cause of endodontic treatment failure due to inadequate sealing ability of root canal sealers. Therefore, it is of interest to evaluate the microleakage of five different root canal sealers using stereomicroscope. Seventy extracted premolars were divided into five groups (n=14) and obturated using respective sealers. Bioactive glass sealer showed lowest microleakage (1.6400 ± 0.3908 mm), while zinc oxide eugenol showed highest microleakage (5.8200 ± 0.6820 mm). Differences were statistically significant (p<0.0001), bioactive glass and bioceramic sealers demonstrated superior sealing ability compared to conventional sealers. Thus, data shows the enhanced sealing efficacy of contemporary bioactive glass and bioceramic sealers compared to traditional root canal sealers, underscoring their potential to improve long-term endodontic treatment results by minimizing microleakage.

## Background:

Successful endodontic treatment depends on effective cleaning, shaping and obturation of the root canal system. The primary objective of obturation is to achieve a hermetic seal that prevents reinfection by microorganisms and penetration of tissue fluids. Gutta-percha is the most widely used obturating material, but it lacks adhesive properties and cannot bond directly to dentinal walls. Therefore, root canal sealers are essential to fill voids and create a seal between gutta-percha and canal walls, thereby improving the overall sealing ability of the root canal filling [1]. Microleakage refers to the passage of bacteria, fluids and toxins between the filling material and dentinal walls. It is considered one of the most important causes of endodontic treatment failure, leading to reinfection and periapical inflammation. Even minimal leakage can compromise treatment success and result in persistent infection. Therefore, the sealing ability of root canal sealers plays a crucial role in determining the long-term prognosis of root canal therapy [2]. Zinc oxide eugenol-based sealers have been widely used due to their antimicrobial properties and ease of handling. However, they have limitations such as solubility, shrinkage and lack of adhesion to dentin. Calcium hydroxide-based sealers were introduced due to their antibacterial properties and ability to stimulate tissue healing, but their sealing ability has shown inconsistent results. Resin based sealers offer improved adhesion and dimensional stability compared to conventional sealers [3]. Recently, newer generation sealers such as bioceramic and bioactive glass-based sealers have been developed. These materials are biocompatible and hydrophilic, allowing better adaptation to dentinal walls. Bioceramic sealers can form chemical bonds with dentin, improving sealing ability. Bioactive glass sealers can form hydroxyapatite upon contact with tissue fluids, enhancing sealing properties and promoting biological compatibility [4]. Various methods are available to evaluate microleakage, including dye penetration, fluid filtration and bacterial leakage techniques. Dye penetration method using stereomicroscope is widely accepted due to its simplicity, accuracy and reproducibility. It allows precise measurement of dye penetration and comparison of sealing ability among different materials [5, 6]. Therefore, it of interest to evaluate the microleakage of zinc oxide eugenol based, resin based, calcium hydroxide based, bioceramic based and bioactive glass-based sealers using stereomicroscope.

## Methodology:

## Study design and sample selection:

This *in vitro* experimental study was conducted on seventy extracted human single-rooted premolars. Teeth extracted for orthodontic or periodontal reasons were included in the study. Teeth with fractures, resorption, incomplete root formation, previous endodontic treatment, or anatomical abnormalities were excluded. All teeth were cleaned using ultrasonic scalers to remove soft tissue debris and calculus and stored in normal saline until use.

## Sample preparation:

The crowns of all teeth were removed using a diamond disc under water cooling to obtain a standardized root length of 15 mm. Access cavities were prepared using endodontic access burs. Canal patency was established using size 10 K-file. Working length was determined and established 1 mm short of apex.

## Root canal preparation:

Biomechanical preparation was performed using proTaper rotary system up to F3 file using crown-down technique. Irrigation was performed using 3% sodium hypochlorite after each instrumentation. Final irrigation was done using 17% EDTA followed by saline to remove smear layer. Canals were dried using paper points.

## Group allocation and obturation:

Samples were randomly divided into five groups (n=14 each):

Group I - Zinc oxide eugenol-based sealer

Group II - Resin based sealer

Group III - Calcium hydroxide-based sealer

Group IV - Bioceramic based sealer

Group V - Bioactive glass-based sealer

Obturation was performed using single cone gutta-percha technique with respective sealers.

## Dye penetration procedure:

Two coats of nail varnish were applied to root surfaces except apical 2 mm. Samples were immersed in 1% methylene blue dye for 72 hours. Teeth were then washed, nail varnish was removed and roots were sectioned longitudinally.

## Microleakage evaluation:

Dye penetration was measured using stereomicroscope at 30x magnification. Microleakage was recorded in millimeters. Scores were also assigned based on dye penetration.

## Statistical analysis:

Data were analyzed using SPSS software version 25.0. Mean and standard deviation were calculated. One-way ANOVA was used to compare groups. Tukey post-hoc test and Chi-square test were applied. Statistical significance was set at p<0.05.

## Results:

A total of 70 teeth were included in the study, with 14 samples in each group. Microleakage was evaluated using dye penetration measured under stereomicroscope. Zinc oxide eugenol-based sealer showed the highest microleakage with a mean value of 5.8200 ± 0.6820 mm, indicating poor sealing ability. Calcium hydroxide-based sealer showed mean microleakage of 4.6793 ± 0.6210 mm, which was lower than zinc oxide eugenol but still relatively high. Resin based sealer demonstrated mean microleakage of 3.4200 ± 0.5502 mm, indicating moderate sealing ability as seen in Table 1. Bioceramic based sealer showed significantly lower microleakage with mean value of 2.3107 ± 0.4803 mm. Bioactive glass-based sealer showed the least microleakage with mean value of 1.6400 ± 0.3908 mm, indicating superior sealing ability. One-way ANOVA showed highly significant difference among groups (F=124.3746, p<0.0001) (Table 1).Post-hoc Tukey test showed significant differences between all groups. Bioactive glass sealer showed significantly lower microleakage compared to all other sealers (Table 2). Score distribution analysis showed that most samples in zinc oxide eugenol group had severe leakage. Bioactive glass and bioceramic sealers showed minimal leakage (Table 3).Bioactive glass sealer showed 71.82% reduction in microleakage compared to zinc oxide eugenol sealer.

## Discussion:

The present *in vitro* study evaluated and compared the sealing ability of zinc oxide eugenol based, calcium hydroxide based, resin based, bioceramic based and bioactive glass-based root canal sealers using stereomicroscopic dye penetration method. Microleakage remains one of the most important factors influencing the long-term success of endodontic treatment, as inadequate sealing can allow bacterial penetration, reinfection and eventual treatment failure. The findings of this study demonstrated statistically significant differences among all groups, with bioactive glass-based sealer showing the least microleakage and zinc oxide eugenol-based sealer showing the highest microleakage [7]. Zinc oxide eugenol-based sealer demonstrated the highest mean microleakage (5.8200 ± 0.6820 mm), indicating poor sealing ability compared to all other sealers evaluated. This finding can be attributed to its lack of chemical adhesion to dentinal walls and its tendency to undergo dimensional changes over time [8]. Zinc oxide eugenol sealers primarily rely on mechanical interlocking rather than chemical bonding, which makes them more susceptible to gap formation. Additionally, their relatively higher solubility in tissue fluids may contribute to gradual degradation and increased leakage. Similar findings were reported by Haji *et al.* [9] and De Angelis *et al.* [10], who observed inferior sealing ability of zinc oxide eugenol-based sealers compared to newer materials, attributing this to their poor adhesion and dimensional instability. Calcium hydroxide-based sealer showed moderate microleakage (4.6793 ± 0.6210 mm), which was lower than zinc oxide eugenol but significantly higher than resin, bioceramic and bioactive glass-based sealers. Although calcium hydroxide sealers offer biological advantages such as antimicrobial activity and stimulation of periapical healing, their sealing ability is limited due to weak bonding with dentinal walls. These sealers lack the ability to form a strong adhesive interface with dentin, which can result in microgaps and increased leakage. Batra *et al.* [11] and Araghi *et al.* [12] also reported higher microleakage with calcium hydroxide-based sealers, suggesting that their limited adhesion contributes to inferior sealing performance. Resin based sealer demonstrated improved sealing ability, with mean microleakage of 3.4200 ± 0.5502 mm. This improvement can be attributed to its adhesive properties and better adaptation to canal walls. Resin sealers can penetrate dentinal tubules and form micromechanical retention, which enhances the seal and reduces leakage. Additionally, their dimensional stability contributes to maintaining the integrity of the seal over time. Kumar *et al.* reported similar findings, concluding that resin-based sealers provide superior sealing ability compared to conventional sealers due to their improved adhesion and physical properties [13]. Bioceramic based sealer showed significantly lower microleakage (2.3107 ± 0.4803 mm), indicating excellent sealing ability. This superior performance may be attributed to its hydrophilic nature, which allows it to utilize moisture present within dentinal tubules for setting and bonding. Bioceramic sealers are capable of forming chemical bonds with dentin through the formation of hydroxyapatite, resulting in improved sealing ability. Furthermore, their excellent dimensional stability and minimal shrinkage contribute to maintaining an effective seal. Mahdi *et al.* reported that bioceramic sealers demonstrate superior sealing ability due to their ability to chemically bond with dentin and form a stable interface [14]. Bioactive glass-based sealer demonstrated the least microleakage (1.6400 ± 0.3908 mm), indicating the best sealing ability among all tested sealers. This exceptional performance may be attributed to its bioactive properties, particularly its ability to form hydroxyapatite upon contact with tissue fluids. This hydroxyapatite formation promotes chemical bonding with dentinal walls and enhances sealing ability. Additionally, bioactive glass sealers exhibit excellent biocompatibility and dimensional stability, which further contribute to their superior performance. Singhal *et al.* reported that bioactive glass sealers can form a mineralized interface with dentin, significantly improving sealing ability and reducing microleakage [15]. The score distribution results also supported these findings, as bioactive glass and bioceramic sealers showed minimal dye penetration in most samples, whereas zinc oxide eugenol-based sealers showed extensive leakage [16]. The percentage reduction analysis further demonstrated that bioactive glass sealer achieved a 71.82% reduction in microleakage compared to zinc oxide eugenol-based sealer, highlighting its superior sealing performance. Overall, the results of this study clearly demonstrate that newer generation sealers such as bioactive glass and bioceramic sealers provide significantly improved sealing ability compared to conventional sealers. Their ability to chemically bond with dentin, form hydroxyapatite and maintain dimensional stability contributes to their superior performance. These findings suggest that the use of bioactive glass and bioceramic sealers may improve the long-term success of root canal treatment by reducing microleakage and preventing reinfection.

## Conclusion:

Bioactive glass-based sealer demonstrated superior sealing ability compared to all other sealers. Bioceramic sealer also showed excellent performance. Conventional sealers such as zinc oxide eugenol and calcium hydroxide showed higher microleakage. The improved sealing ability of bioactive glass and bioceramic sealers may enhance long-term success of endodontic treatment.

## Figures and Tables

**Table 1:** Comparative evaluation of microleakage (mm) among different sealers

**Group**	**Sealer type**	**Mean (mm)**	**Std. Deviation**	**95% CI Lower**	**95% CI Upper**	**F value**	**P value**
Group I	Zinc oxide eugenol	5.82	0.682	5.4263	6.2137	124.3746	<0.0001*
Group II	Resin based	3.42	0.5502	3.1025	3.7375		
Group III	Calcium hydroxide based	4.6793	0.621	4.3208	5.0378		
Group IV	Bioceramic based	2.3107	0.4803	2.0335	2.5879		
Group V	Bioactive glass based	1.64	0.3908	1.4145	1.8655		
*=Significant

**Table 2:** Post-Hoc Tukey test for intergroup comparison of microleakage

**Group comparison**	**Mean difference (mm)**	**Std. Error**	**P value **
ZOE vs Resin	2.4	0.2063	<0.0001*
ZOE vs Calcium hydroxide	1.1407	0.2063	<0.0001*
ZOE vs Bioceramic	3.5093	0.2063	<0.0001*
ZOE vs Bioactive glass	4.18	0.2063	<0.0001*
Resin vs Calcium hydroxide	1.2593	0.2063	<0.0001*
Resin vs Bioceramic	1.1093	0.2063	<0.0001*
Resin vs Bioactive glass	1.78	0.2063	<0.0001*
Calcium hydroxide vs Bioceramic	2.3686	0.2063	<0.0001*
Calcium hydroxide vs Bioactive glass	3.0393	0.2063	<0.0001*
Bioceramic vs Bioactive glass	0.6707	0.2063	0.0118*
*= Significant

**Table 3:** Distribution of microleakage scores among study groups

**Group**	**Score 1 (1-3 mm)**	**Score 2 (3-5 mm)**	**Score 3 (>5 mm)**	**Chi-square value**	**P value**
ZOE	0 (0.00%)	3 (21.42%)	11 (78.57%)	52.68	<0.0001*
Resin	3 (21.42%)	10 (71.42%)	1 (7.14%)		
Calcium hydroxide	1 (7.14%)	8 (57.14%)	5 (35.71%)		
Bioceramic	11 (78.57%)	3 (21.42%)	0 (0.00%)		
Bioactive glass	14 (100.00%)	0 (0.00%)	0 (0.00%)		
*= Significant
